# esyN: Network Building, Sharing and Publishing

**DOI:** 10.1371/journal.pone.0106035

**Published:** 2014-09-02

**Authors:** Daniel M. Bean, Joshua Heimbach, Lorenzo Ficorella, Gos Micklem, Stephen G. Oliver, Giorgio Favrin

**Affiliations:** 1 Cambridge Systems Biology Centre, University of Cambridge, Cambridge, United Kingdom; 2 Department of Biochemistry, University of Cambridge, Cambridge, United Kingdom; 3 Dipartimento di Biochimica, Universita’ degli studi di Pisa, Pisa, Italy; 4 Department of Genetics, University of Cambridge, Cambridge, United Kingdom; University of Cyprus, Cyprus

## Abstract

The construction and analysis of networks is increasingly widespread in biological research. We have developed esyN (“easy networks”) as a free and open source tool to facilitate the exchange of biological network models between researchers. esyN acts as a searchable database of user-created networks from any field. We have developed a simple companion web tool that enables users to view and edit networks using data from publicly available databases. Both normal interaction networks (graphs) and Petri nets can be created. In addition to its basic tools, esyN contains a number of logical templates that can be used to create models more easily. The ability to use previously published models as building blocks makes esyN a powerful tool for the construction of models and network graphs. Users are able to save their own projects online and share them either publicly or with a list of collaborators. The latter can be given the ability to edit the network themselves, allowing online collaboration on network construction. esyN is designed to facilitate unrestricted exchange of this increasingly important type of biological information. Ultimately, the aim of esyN is to bring the advantages of Open Source software development to the construction of biological networks.

## Introduction

**Figure 1:**
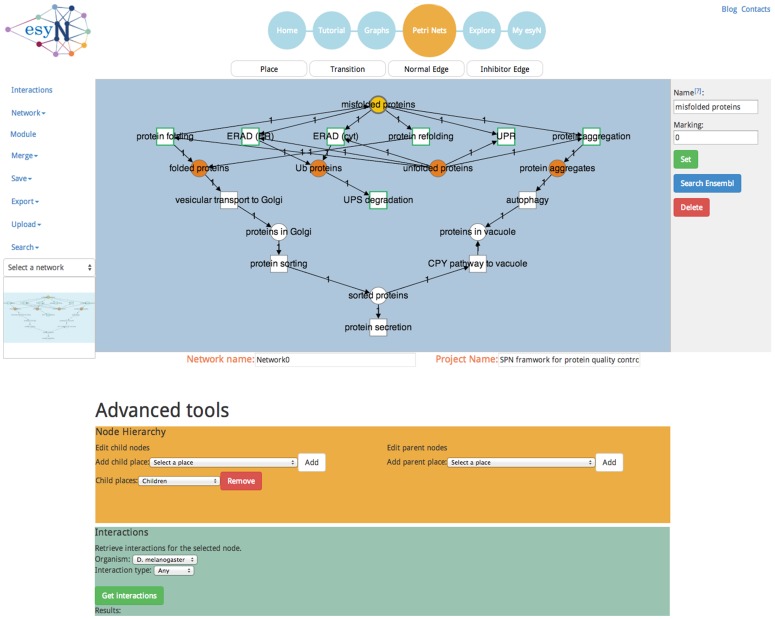
Screenshot of the esyN Model-building tool. The left panel is the menu for the network building tool, with options e.g saving, uploading and exporting projects. The central blue panel is the window in which the network is displayed, in this case a model. This window is used for node and edge creation and selection. The right panel contains tools for editing individual nodes and edges. The lower yellow panel displays options to create parent-child relationships for the selected node. The lower right panel is the interface to the supported InterMine databases, allowing interaction data to be automatically retrieved for the selected node. The page layout is identical for the Graphs tool, which the exception of the node hierarchy panel (yellow), which is absent for graphs.

The advent of large data warehouses that contain interaction data such as BioGRID [1], iRefIndex [2] and IntAct [3] databases is facilitating the study of biological pathways and networks. Building and modeling these networks is an increasingly valuable tool in biological research, particularly as many complex diseases are now thought to be the result of subtle dysregulation of many biological pathways [4], [5]. It is challenging, however, to identify which interactions are relevant to a given biological question. Although biological interaction networks are widely constructed and published, network models are not readily exchanged. We have developed esyN (easy networks, available at: www.esyn.org, Fig. 1) to facilitate the exchange of network data and streamline the process of collaborating on their construction. Unlike the major repositories of biological pathways e.g. KEGG [6] or Reactome [7], anybody is free to create a network and make it publicly available within esyN. In addition, users can easily import any public data, modify it and publish their version.

The large volume of interaction data that is currently available has proven to be extremely valuable in understanding specific processes such as the pathology of a disease. However, it is not enough to simply include all interactions of every gene known to be associated with the disease, mainly because interaction data are typically collected under laboratory conditions, that are unlikely to accurately represent the state of the cellular network in the diseased state. In order to study the network of interactions related to a disease, we need to build a “differential” network [8]; that is, the network of interactions that differ between the healthy and the disease states. Although efforts are under way to directly construct such networks [9], [10], there are no repositories of such differential networks and this means that each researcher needs to start from literature and/or raw data. With esyN, we intend to encourage researchers from any field to share the networks that they have constructed.

The rapidly increasing volume of biological data also aids the development of quantitative, predictive models of biological systems. One relatively straightforward method to generate such a model is a Stochastic Petri Net (SPN) [11]. We have therefore also developed a simple online tool to edit SPN models, which can be shared in the same ways as interaction networks. Any user can import a public model into their own project, thus esyN acts as a repository of Petri Net “modules” that can be re-used elsewhere. In practice, we intend esyN to deliver the equivalent advantages to those of open-source software development to the network modeling community, by enabling useful modules to be freely redistributed and reused. Over time, this will reduce the need for modeling effort to be duplicated within the research community.

esyN allows the building, viewing, sharing and publishing of two types of networks: Graphs (simple directed or undirected graphs) and Petri net models.

## Graphs

Graphs are the simplest way to visualize interaction data, in which nodes represent biological entities (*e.g.* gene, protein, molecule) and the interactions between them are represented by edges connecting nodes.

One of the primary functions of esyN is to provide a public and unrestricted repository for interaction networks. We have created a companion web tool for the construction, viewing, and editing of biological interaction network graphs using cytoscape.js, a javascript library [12] that facilitates the development of graph-centric web applications. To streamline the process of constructing interaction networks, we have added integration with the InterMine framework [13]–[15] using the imjs library [15]. This allows interaction data to be retrieved for human [16], yeast [17] and fruitfly [18] networks. In this way, users are able to interactively build up interaction networks using data from public resources. When edges are imported via InterMine databases, the supporting references are also automatically associated with the edge (references can also be added manually). In addition, users can upload interaction data in spreadsheet format, or export data from existing Cytoscape projects in JSON format [19].

We have used our online tool to create interaction networks for genes related to Alzheimer’s disease, Parkinson’s disease, and Amyotrophic Lateral Sclerosis. Starting from genes currently linked to each disease, we built up each network including only those edges related to the disease state (based on literature [20]–[29]) rather than simply including all interactors, not all of which will be relevant to the disease. The rationale for including or excluding nodes is also given in the description field for the project. By making these networks publicly accessible in the esyN database, we have provided a starting point for any researchers wishing to study the interaction networks of these diseases. Other researchers are free to contribute their own versions of these networks, which may differ from ours. It is our intention to promote and enable unrestricted sharing of any such network, encouraging more widespread collaboration and exchange of information.

## Petri Nets

**Figure 2:**
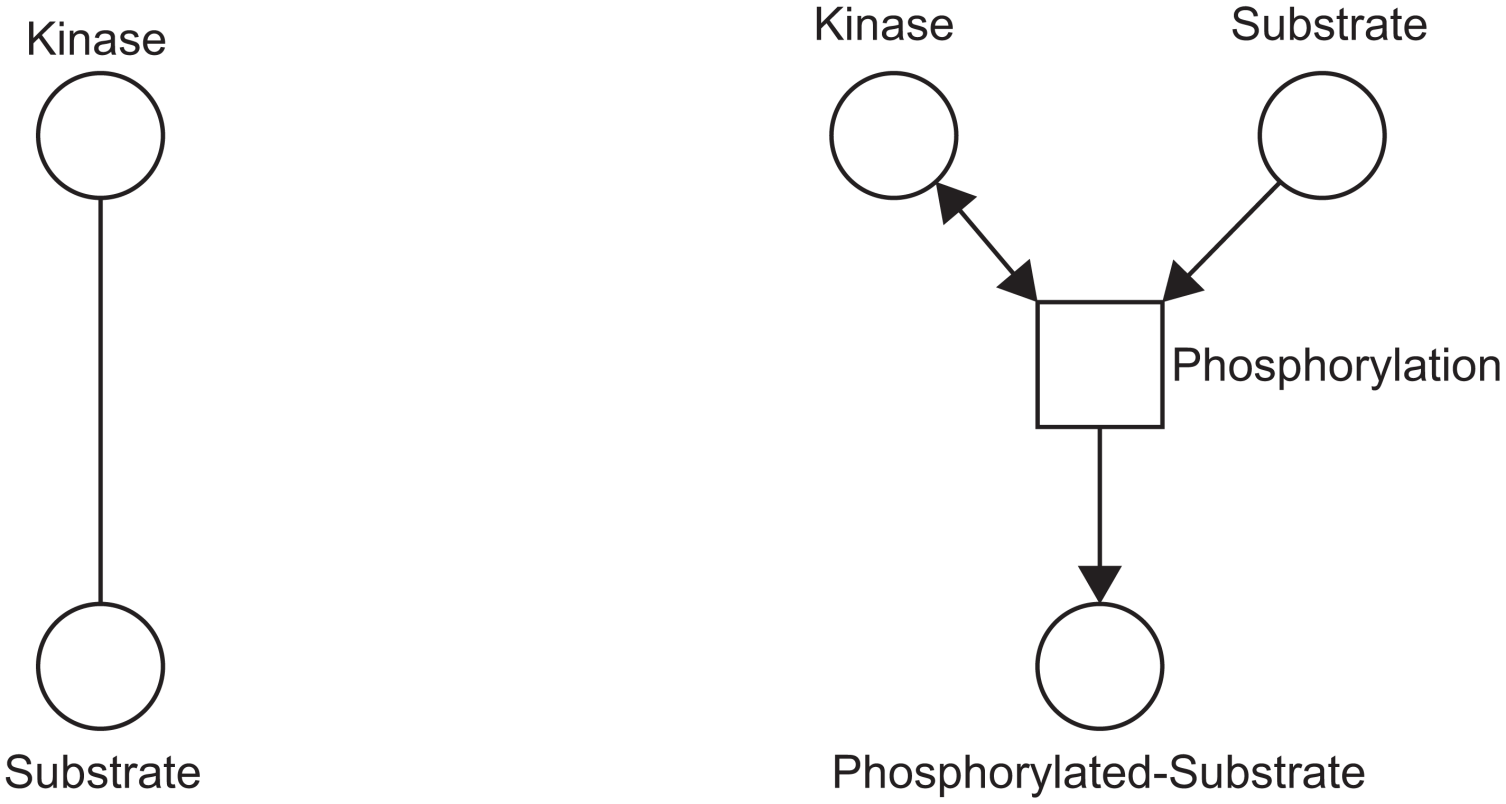
Schematic representation of Kinase-Substrate interaction in a graph (left) and Petri Net (right). Additional nodes and edges are required to represent the process in the Petri Net framework.

Stochastic Petri Nets (SPN) represent a straightforward graphical language to describe stochastic processes. We have chosen to use SPN to build our models because of their relative simplicity, which makes SPN the ideal formalism for creating complex models using smaller ones as building blocks. In contrast to graphs, Petri nets depict reactions in much more detail (Fig. 2), and therefore it is not always straightforward to map protein - protein interaction networks onto a Petri net model. Petri Net models represent a mathematical model of a dynamic process, whereas graphs are a static representation of interactions. Petri Nets require much more parameterization than graphs (for example the amount of each species and the stoichiometry of reactions), which is often a major obstacle to their construction. The interactions that make up graphs are routinely measured on a large scale for many organisms, whereas the same is not possible for Petri Nets. Importantly Petri nets can be converted into a series of matrices, which can then be used for simulations in which a series of transitions “fire”, moving tokens between places. For simulations, SPN models can be converted into matrices and downloaded in JSON format. We have developed an R script for running simulations using the Gillespie algorithm, of the Petri net models created with esyN, and this can be found at: http://github.com/esyN/esyN-simulation.

Just as is the case for network graphs, esyN provides a simple web tool for viewing and editing Petri Nets. We used this tool to construct a number of basic Petri net models of pathways using data from Reactome [7] and from the literature [25], [30]–[37]. We have made these models public (accessible at http://www.esyn.org/browse.php), thus enabling their use as “building blocks” for the construction of other models. With time, the esyN collection of pathways will grow allowing the user to build networks progressively more easily.

In addition to these models, we have created a series of small functional units (templates or modules) designed to speed up the creation of Petri Net models by making frequently used structures (e.g. simple logic gates) available for any user to quickly add to their projects.

The ability to build network graphs using experimental interaction data (both physical and genetic) retrieved from InterMine is meant to facilitate and guide the construction of the corresponding Petri net models which have a similar blueprint but every interaction has to be modeled individually (Fig. 2). Each transition node in the network can be associated with one or more references, supporting its inclusion in the network, or the structure of the edges connected to it.

## Comparison to Other Tools

The esyN web tools for constructing networks are not designed to replace existing tools such as Cytoscape [38] or Snoopy [39]; rather, they are designed to complement these much more extensive tools, providing a fast and convenient interface to the network repository. We encourage users wishing to perform more extensive analyses to download the network data and use an established tool such as Cytoscape. For this reason, we allow all network data to be downloaded in standard formats (comma-separated values for interaction networks, JSON format matrices for Petri net models).

There is a large variety of repositories of biological pathways and models (e.g. Kegg [6], Reactome [7], BioCarta [40], BioModels [41]). esyN is intended to be complementary to these databases, allowing fast, unrestricted exchange of users’ networks that have been constructed using data from a variety of sources. Importantly, once published these networks can be copied and re-used freely by the community in a similar fashion to Open Source software.

In short esyN is a unique tool as it integrates tools for the creation of network models with an open repository that stores them. This allows public models to be used as building blocks for further models. In Table S1 we have listed a more complete list of comparable tools with their relative features.

## Methods

### Software and Libraries

esyN is primarily written in the javascript language, using the following libraries: cytoscape.js [12], intermine [13], jQuery [42], angularJS [43], underscore.js [44]. The network database uses MySQL. Hosting is provided by the University of Cambridge. All source code is available under the LGPL license at: https://github.com/esyN/code.

### Requirements

esyN has been tested on Google Chrome (version 34), Mozilla Firefox (version 29), Safari (version 7.0.3), Internet Explorer (version 11) and Opera (version 20). esyN requires a modern web browser. To save projects online, publish projects, and collaborate on projects, users must log in. Registration is free and only requires an email address. We use persona [45] for authentication, meaning we do not store users’ passwords. No other features of esyN require users to log in.

### Constructing Graphs

Graphs consist of nodes connected by edges, which can be directed or undirected. Each edge can also have a type (e.g. “genetic” or “physical”). Nodes in a graph may represent physical entities such as genes or proteins, or may themselves contain a nested network. Interaction data can be automatically imported from FlyMine [18], YeastMine [17] and MetabolicMine [16]. Graphs can be created from interaction data uploaded as comma-separated values, or as JSON [19] data exported from Cytoscape.

### Constructing Petri nets

Petri Net models are bipartite directed graphs. For an in-depth background on Petri nets, see [46]. Briefly, nodes can be either “places” representing entities that can be quantified (by the number of tokens they contain), or “transitions” representing actions that act on the places to change their quantities. Places may represent real physical entities, or they can themselves contain other places (these are called “coarse places”). Edges connecting coarse places to transitions represent a process that happens to every one of the contained places. Transitions can contain nested networks as a way to hierarchically organize a large project. Related nodes can be found using FlyMine [18], YeastMine [17] and MetabolicMine [16] to find interacting genes or proteins. Models can also be uploaded from an existing project created using Snoopy.

### Collaboration

From their own home page (www.esyn.org/home.php), users can set the properties of each of their projects. There are two different types of collaborator: 1) “Viewers” are able to open and view a project, but cannot make changes (unless they save their own copy). Viewers can only see the most recent version of a project. 2) “Editors” are able to make changes to a project. Collaborators are added by email address, which must be the address used to log in.

### Publishing

Any user can make any project public at any time. When a project is made public, it is copied into a separate database. This allows the user to continue working on their own project without affecting the version they made public. Indeed, nobody is able to directly edit public projects. Any user is free to import any public project into their own workspace, where they are able to modify their copy and, if they choose to, make their version public.

## Supporting Information

Table S1 **List of tools comparable to esyN and their relative features.**
(XLS)
