# Correction: esyN: Network Building, Sharing and Publishing

**DOI:** 10.1371/journal.pone.0204058

**Published:** 2019-01-09

**Authors:** Daniel M. Bean, Joshua Heimbach, Lorenzo Ficorella, Gos Micklem, Stephen G. Oliver, Giorgio Favrin

In the Funding section, the grant number from the funder Wellcome Trust/MRC is listed incorrectly. The correct grant number is: 089703/Z/09/Z, 099133/Z/12/Z.
